# Digital droplet PCR analysis of organoids generated from mouse mammary tumors demonstrates proof-of-concept capture of tumor heterogeneity

**DOI:** 10.3389/fcell.2024.1358583

**Published:** 2024-05-15

**Authors:** Katherine E. Lake, Megan M. Colonnetta, Clayton A. Smith, Kaitlyn Saunders, Kenneth Martinez-Algarin, Sakshi Mohta, Jacob Pena, Heather L. McArthur, Sangeetha M. Reddy, Evanthia T. Roussos Torres, Elizabeth H. Chen, Isaac S. Chan

**Affiliations:** ^1^ Department of Internal Medicine, Division of Hematology and Oncology, University of Texas Southwestern, Dallas, TX, United States; ^2^ Harold C. Simmons Comprehensive Cancer Center, University of Texas Southwestern Medical Center, Dallas, TX, United States; ^3^ Department of Molecular Biology, University of Texas Southwestern, Dallas, TX, United States; ^4^ Hamon Center for Regenerative Science and Medicine, University of Texas Southwestern Medical Center, Dallas, TX, United States; ^5^ Department of Cell Biology, University of Texas Southwestern Medical Center, Dallas, TX, United States; ^6^ Division of Medical Oncology, Norris Comprehensive Cancer Center, Keck School of Medicine, University of Southern California, Los Angeles, CA, United States

**Keywords:** heterogeneity, metastasis, breast cancer, ddPCR, FGFR1

## Abstract

Breast cancer metastases exhibit many different genetic alterations, including copy number amplifications (CNA). CNA are genetic alterations that are increasingly becoming relevant to breast oncology clinical practice. Here we identify CNA in metastatic breast tumor samples using publicly available datasets and characterize their expression and function using a metastatic mouse model of breast cancer. Our findings demonstrate that our organoid generation can be implemented to study clinically relevant features that reflect the genetic heterogeneity of individual tumors.

## 1 Introduction

While breast cancer is the most prevalent cancer among women, most patients are diagnosed with early-stage breast cancer and cured by multi-modality treatment (Siegel et al., 2022). However, around 10% of patients will develop metastatic breast cancer (MBC), which is the main driver of breast cancer related deaths (Scully et al., 2012; Esposito et al., 2021). Although breast cancer survival rates have substantially improved over 20 years, that is largely attributed to increased screening and improved adjuvant therapies (Nolan et al., 2023). However, the same improvements in survival have not been seen among patients with metastatic breast cancer (Hashim et al., 2016). A reason for therapeutic resistance of MBC is partly due to the relative lack of targetable genetic vulnerabilities that act as intrinsic mediators of breast cancer cell metastasis. Recent literature suggests that cancer cell metastasis is defined by copy number alterations and not sufficiently by genetic mutations alone (Zack et al., 2013; Martelotto et al., 2017; Siegel et al., 2018; Priestley et al., 2019). Recent and early literature have suggested that metastatic events are spurred by only a small number of cells from genetically heterogeneous primary tumors, and that only a few genetically predisposed cells are capable of metastasis (Fidler, 1978; Merino et al., 2019). Both intratumoral and intertumoral heterogeneity has been cited as the largest roadblock to the development of individualized therapy (Bedard et al., 2013; Dagogo-Jack and Shaw, 2018; LeSavage et al., 2022; Xu et al., 2024). However, the functional value of heterogeneity within tumors has been hard to model using traditional cell lines and mouse models (Dai et al., 2017; Pasha and Turner, 2021; Gui and Bivona, 2022). Patient-derived organoid-based methods provide an ideal and representative platform to study human tumor heterogeneity (Drost and Clevers, 2018). They model morphologic and structural properties of the original tumor and also mirror the tumor’s epigenetic, phenotypic, and metabolic diversity. Our group and others have used organoid-based platforms to model cell-cell signaling and interactions with the tumor microenvironment (Hwang et al., 2019; Chan and Ewald, 2022; LeSavage et al., 2022; Hogstrom et al., 2023). Organoids also have been used to accurately predict therapeutic response, suggesting their potential role in personalized medicine (Chan and Ginsburg, 2011; Vlachogiannis et al., 2018; Larsen et al., 2021; Guillen et al., 2022; Wang et al., 2023). These strengths set them apart from traditional clonal breast cancer cell lines, which do not specifically capture the genetic diversity in breast cancer and do not recapitulate individual patient’s tumor microenvironment (Sharma et al., 2010). Furthermore, patient-derived organoids are useful models to use for pre-clinical drug screening given that they can accurately predict clinical outcomes (van de Wetering et al., 2015). In this study, we show it is technically feasible to evaluate copy number (CN) heterogeneity of tumors and organoids at various resolutions, including pooled organoid samples, single organoids, and invading organoids, using clinically relevant target genes.

## 2 Results

### 2.1 Organoid generation retains clinically relevant tumor genomic heterogeneity

**FIGURE 1:**
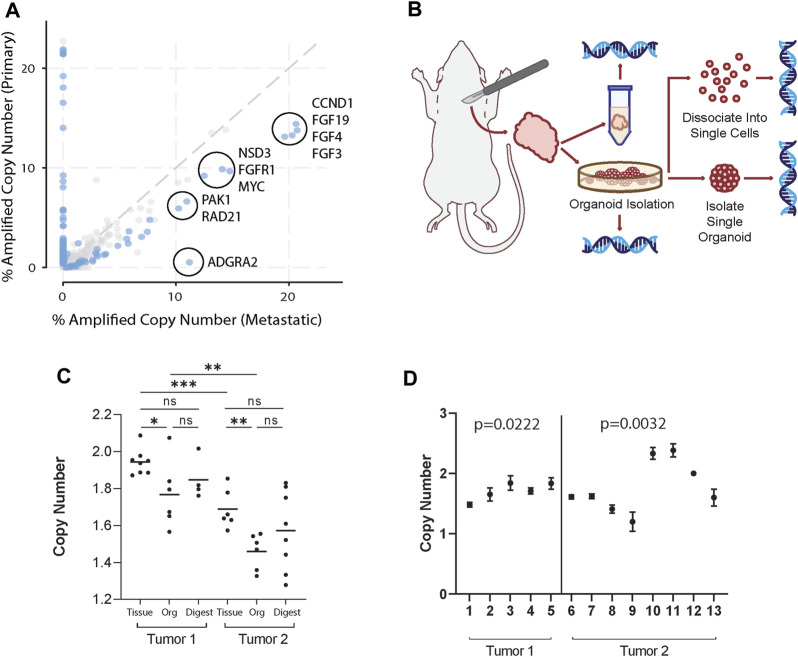
Organoid generation retains clinically relevant tumor genomic heterogeneity. **(A)** Copy number amplified genes in IDC patients found in distant organ metastasis, unspecified metastasis site, or lymph node metastasis compared to primary breast cancer samples in the GENIE database. Of the genes (dots) amplified in greater than 10% of primary or metastatic samples, the top 10 statistically significant (blue dots) differentially amplified in metastatic samples are circled. These include ADGRA2, RAD21, PAK1, FGF4, NSD3, FGF19, FGF3, CCND1, FGFR1, and MYC. **(B)** Schema of workflow for sample generation. Large primary mammary tumors are dissected from the fat pad. Tissue segments are excised from 4 distinct regions of the tumor to ensure adequate sampling. Organoids generated from the tumors were sampled and dissociated and strained to single cells, taken as pooled organoid samples, or isolated to single organoids. All samples were then used to isolate gDNA and perform ddPCR. **(C)** FGFR1 copy numbers in tissue, pooled organoids (Org), and single cell digests (Digest) from two different mice normalized to the housekeeping gene RPP30. Tissue sample copy number alterations are statistically different than pooled organoid samples (Mann-Whitney, *p* < 0.05). Each point represents separate batches of organoids generated from primary tumor (*n* = 2, *r* = 2). Each primary tumor was split into 4 samples for separate organoid generation to ensure adequate sampling over the whole tumor. **(D)** FGFR1 copy numbers in single organoids from tumors from two different mice. Among each tumor there exist statistically distinct copy numbers (Kruskall-Wallis, *p* = 0.0222; *p* = 0.0032).

To identify potential CNA enriched in metastatic breast cancer, we used the Project GENIE database (Pugh et al., 2022) and cBioPortal (Cerami et al., 2012). For analysis, genes with copy number alterations in invasive ductal carcinoma (IDC) patient samples were selected, as it is the most common histologic class of breast cancer in humans. Genes were filtered first by copy number amplification in >10% of metastatic IDC samples, defined as having distant organ metastasis, unspecified metastasis site, or lymph node metastasis. While these sites of metastasis have distinct biology, the purpose of this study aims to provide a framework to study how genetic heterogeneity at a primary tumor site may be reflected in the metastatic process. Thus, primary tumor samples were compared against all other metastatic samples grouped together. From those, the top 10 differentially amplified genes in metastatic over primary samples were identified. In order by logarithmic ratio, they include ADGRA2, RAD21, PAK1, FGF4, NSD3, FGF19, FGF3, CCND1, FGFR1, and MYC (Figure 1A; Supplementary Table S1).

We then hypothesized that in a primary tumor there may be a heterogeneous mix of cancer cells highly copy number amplified in select genes as well as those that are not. In order to study the genetic heterogeneity of mammary tumors using the genes identified in Figure 1A, we chose to utilize the PyMT mouse model, due to its similarity to IDC evolution in humans and high potential for metastasis [(Lin et al., 2003; Attalla et al., 2021)]. Further, recent whole genome sequencing of the MMTV-PyMT model characterized key copy number alterations (Rennhack et al., 2019). We then identified genes that both were overexpressed in metastatic samples over primary breast cancer and were also altered in the MMTV-PyMT mouse model (Rennhack et al., 2019). From the top 10 differentially expressed genes, we found four genes that fit these criteria (Rennhack et al., 2019). These genes include ADGRA2, FGFR1, NSD3, and PAK1.

Next, we generated mammary tumor organoids derived from MMTV-PyMT mice using differential centrifugation (Chan et al., 2020; Chan and Ewald, 2022; Cornelius et al., 2022). Using this approach, organoids are generated by digesting the entire tumor tissue, and thus could represent its overall genetic heterogeneity. To demonstrate our organoid generation method captures intratumoral heterogeneity, we processed mouse mammary tumors and collected genomic DNA (gDNA) from whole tumor tissue, pooled organoids generated from tumor tissue, single cell digests of organoids, and single organoid samples (Figure 1B). Digital droplet PCR (ddPCR) was then used to identify the copy number of FGFR1, ADGRA2, NSD3, and PAK1 within each collected sample compared to reference gene RPP30. Notably, in all but PAK1, tumor tissue copy number was significantly increased from pooled organoid samples (Mann-Whitney, *p* < 0.05) (Figure 1C; Supplementary Figures S2–S4). A potential reason could be that pooled organoid samples include only malignant mammary epithelium and exclude any stroma, muscle, or other cells present in tissue samples (Chan and Ewald, 2022; Cornelius et al., 2022). Thus, CN reads from tumor tissue would be closer to a diploid state. Interestingly, cancerous organoids have an overall copy number deleted state in select genes. While it was unsurprising that they were not copy number amplified given they are derived from primary tumor samples, future studies may examine the significance of a heterogeneous copy number deleted state. Additionally, no single cell digest copy number was statistically distinct from the pooled organoid sample from the same tumor in any tested gene. However, single cell digests lose the potential for functional testing and risk skewed genetic profiles through imperfect digestion or straining.

Interestingly, pooled organoid samples differed from a normal diploid state, suggesting the sample was heterogeneous and included cells with CN variants reflective of an aneuploid state (i.e., CN 0 or 1 or 3). To identify whether this heterogeneity was consistent at the organoid level, we isolated gDNA from single organoids and analyzed the CN of ADGRA2, FGFR1, NSD3, and PAK1. CN of select genes differed widely among individual organoids (Figure 1D; Supplementary Figures S2–S4). Additionally, in single organoids, CN was rarely identified as exactly 2 (normal diploid state), suggesting: 1) organoids are genetically distinct from each other, and 2) they also must contain cells with CN alterations in different proportions. In summary, our single organoid isolation method captures both the intra- and inter-organoid genetic heterogeneity in tumor samples.

### 2.2 FGFR1 is copy number amplified in both human metastasis and *in vitro* model of metastasis

**FIGURE 2:**
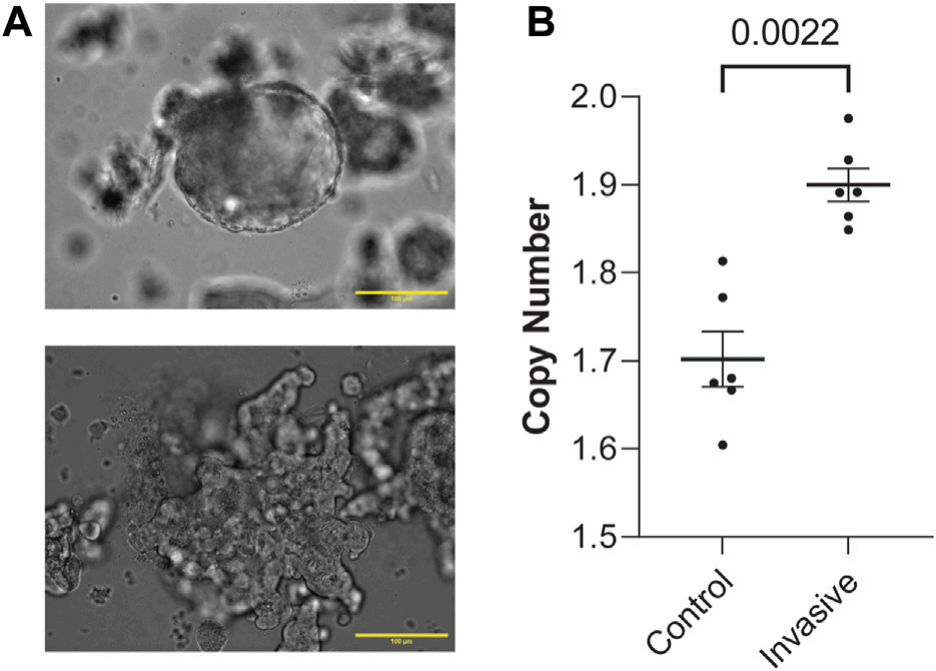
FGFR1 is copy number amplified in both human metastasis and *in vitro* model of metastasis. **(A)** Representative image of a non-invading, control group organoid in liquid media (top). Representative image of an invading, experimental group organoid post-collagenase digestion in liquid media (bottom). Both of these organoids were initially allowed to grow either in media (control) or type-1 collagen ECM (experimental) for 48 h. These images are representative of the conditions assessed in this figure **(B)**. **(B)** FGFR1 copy number is amplified in invading organoids vs. control organoids from PyMT mouse primary mammary tumor samples (Mann-Whitney, *p* = 0.0022). Each point represents copy number measured from duplicate control or invasive organoid samples from 2 PyMT mouse primary mammary tumor organoids. Duplicates were performed for each sample in ddPCR. Duplicates were not included for those >20% apart.

Next, to determine whether these genes are involved in metastasis, we generated organoids from MMTV-PyMT mice and randomly split them into a control group grown in suspension and an experimental group grown in 3D culture. Growing organoids in 3D culture allows *ex vivo* assessment of their invasive potential through collective migration into collagen (Chan et al., 2020; Chan and Ewald, 2022; Cornelius et al., 2022). Organoids were either kept in media suspension (Figure 2A) or embedded in 3D collagen I gels and assessed for invasion (Supplementary Figure 1B). Both the control and experimental groups were allowed to grow for 48 h. Then, we digested the ECM in the experimental group and isolated genomic DNA from both groups. Interestingly, invasive organoids retain their morphology after removal from collagen gels (Figure 2A; Supplementary Figure 1C). Of the genes enriched in metastatic sites from Figure 1A, FGFR1 is the most clinically mature target (Babina and Turner, 2017). We reasoned if FGFR1 CN is amplified in metastatic lesions, it could potentially be amplified starting at the earliest stages of metastasis, invasion out of the primary tumor. To test whether FGFR1 copy number amplification is associated with invasion, we determined gene CN of FGFR1 in control and invasive samples using ddPCR. We found that invasive organoids have statistically significant CNA compared to control organoids (Mann-Whitney, *p* = 0.0022) (Figure 2B). These findings demonstrate that higher FGFR1 CNA correlates with organoid invasion, suggesting that FGFR1 is heterogeneously expressed in the earliest stages of metastasis in addition to the developed metastases. Although we observed increased CN of FGFR1 in invasive organoids over non-invasive organoids, the average CN of each condition was below 2. This finding suggests cells with FGFR1 CN deletion could also be present at the primary tumor site. Additionally, the average CN of each condition was not an integer value, indicating heterogeneity of FGFR1 alterations between organoids in the sample, as a sample of uniform organoids would have copy number of 0, 1, 2, 3, and so on. While it is possible that cells with CN deletions are dying in the invasive condition, it is also possible that FGFR1 copy number amplified cells are expanding in the invasive condition.

## 3 Discussion

In this study, we demonstrate proof-of-concept for ddPCR analysis of tumor organoids, cell suspensions, and single cells. We determine ten copy number amplifications enriched in metastatic site tumors over primary breast tumors using Project GENIE (Pugh et al., 2022). In assessing FGFR1, we found that it is amplified in invading organoids, suggesting the importance of FGFR1 in the early stages of metastasis. Lastly, given the increasing clinical importance of cancer epithelial heterogeneity within the breast tumor, we demonstrate that these methods of organoid generation and isolation capture intratumoral genetic diversity between individual organoids. As far as we know, this is the first study to analyze the copy number state of individual breast organoids. Further, one main challenge to using organoids in preclinical models is genetic drift (Lo et al., 2020). Here we show that generating and isolating organoids without passaging captures both intratumoral heterogeneity and reduces the chance of genetic drift as organoids are used soon after tumor digestion. Given that functional models are lacking in the literature to test the impact of tumor heterogeneity on tumor metastasis (Lawson et al., 2018) and immune interactions (Xu et al., 2022), this model could be useful for further experimentation. Using ddPCR on our organoid models could be used to assess tumor genomic heterogeneity and evaluate therapeutic response and resistance. For example, patient derived organoids collected at different time points could be used to identify moments of acquired resistance and inform therapeutic decision-making.

This work is limited by the number of tumors evaluated and the number of genes experimentally validated. In addition, only 1 mouse model was used to assess copy number heterogeneity, which may differ from other mouse models of breast cancer and also from human tumors. Future studies should aim to evaluate and validate CNAs in human organoid samples and additional animal models to uncover additional mechanisms of metastasis and therapeutic resistance. Further, single organoids are typically comprised of 50–100 cells, and thus the quantity of gDNA extracted is limited, restricting the replicate number, positive droplets in ddPCR, and ultimately statistical power of the analysis. Additionally, several studies have described genetic drift of organoids in long-term culture environments (Sachs et al., 2018; LeSavage et al., 2022). In this study, we assessed organoid CNAs without passaging, however organoid CNAs could evolve over time through long-term culture and passaging, as previously described (Sachs et al., 2018). Future work is needed to refine the appropriate use-case for each organoid culture method.

Our results also provide a potential method to functionally validate the role of FGFR1 in metastatic development. The implications of FGFR1 manipulation should be assessed in both *in vitro* tumor organoid models as well as *in vivo* metastasis models to test the necessity and sufficiency of FGFR1 in the metastatic cascade. Of the genes evaluated in this study, therapeutic agents targeting FGFR1 and FGFR4 are the most clinically mature. As of 5 April 2024, ClinicalTrials.gov lists 105 trials evaluating FGFR inhibitors for the treatment of various cancers, including breast cancer (Katoh et al., 2024). Furthermore, alterations in FGFR family genes in cancer are considered potential biomarkers for therapeutic response to tyrosine kinase inhibitors (TKIs) (Liu et al., 2020). In the future, using ddPCR on patient-derived organoids to assess FGFR1 expression could help predict responsiveness to TKI or novel FGFR inhibitors. The organoid-based workflow described in our paper could also be used to validate other targets identified in our screen. Overall, our work contributes to the growing need for improved modeling of intratumoral cancer epithelial cell heterogeneity, which has broad implications on clinical practice and cancer biology.

## 4 Materials and methods

### 4.1 Copy number amplifications in metastatic over primary breast cancers

We analyzed the GENIE Cohort v13.0-public dataset from invasive ductal carcinomas (IDC) tumors. We selected all samples for IDC, then stratified based on primary vs. metastatic sample. We obtained copy-number amplification (CNA) information from metastatic samples defined as distant organ metastasis, unspecified metastasis site, or lymph node metastasis (2,931 samples) and primary breast tumors (6,254 samples). The alteration frequency was analyzed by using cBioPortal.

### 4.2 Animals, tumor, organoid, and cell samples

11–14-week-old MMTV-PyMT mice with large (>0.5 cm) palpable mammary tumors were identified. Mice were sacrificed according to IUCAC guidelines with CO2 asphyxiation and secondary cervical dislocation. Tumors were dissected and organoids generated following the protocol described previously (Cornelius et al., 2022). For Figure 1A, tumors were dissected into quadrants, and samples taken as described in schema. Single cells were strained and harvested after Tryple™ Express (Gibco™; cat: 12605036) digestion and visual verification of single cell dissociation. Unless performing an invasion assay, organoids and single cells were harvested immediately upon generation and not cultured or passaged. Organoid invasion assay was performed as described previously (Cornelius et al., 2022). Collagenase was used to digest 3D collagen I gels to isolate invasive organoids. Single organoids were isolated from culture using P20 pipettes set to ∼5uL until a single organoid was isolated into a well and visualized via microscope. If organoid density was too high for single organoid isolation, ∼20uL was diluted into 500uL PBS in a 12-w plate. Microscope verification was performed for each single organoid. Genomic DNA was isolated using Quick-DNA Miniprep kit (Zymo Research).

### 4.3 Droplet digital PCR

Primers and probes for ddPCR for reference housekeeping gene (RPP30) and target genes (ADGRA2, FGFR1, NSD3, PAK1) were purchased from Bio-Rad Laboratories (Assay IDs: dMmuCNS822293939, dMmuCNS263266645, dMmuCNS890129559, dMmuCNS681547140, dMmuCNS429051281, respectively). RPP30 was chosen as a reference gene as it is commonly used as a robust reference for quantification of mammalian genomic DNA (Hindson et al., 2011; Mancini et al., 2011; Lin et al., 2016; Imaizumi et al., 2019; Oscorbin et al., 2019; Wen et al., 2021). Genomic DNA (up to 1ng/sample), ddPCR supermix (no dUTP) (Bio-Rad; cat: 1863024), HaeIII restriction enzyme and rCutsmart buffer (NEB; cat: R0108S), and nuclease-free water were mixed with primer/probe for target and reference gene according to manufacturer recommendations. FAM labeled probes were used for target genes and a HEX labeled probe was used for RPP30 to allow both target and reference reads to be determined in the same sample. Droplets were generated using the QX200 droplet generator (Bio-Rad) and subsequently thermocycled according to manufacturer recommendations. Following PCR amplification, droplet read data was acquired using the QX200 droplet reader (Bio-Rad) and analyzed with QuantaSoft software (BioRad). Droplets were plotted based on their fluorescence amplitude of each probe, high in positive droplets and low in negative droplets. Thresholds to determine positive and negative droplets were visually set between the two clusters, with the user blinded to sample identity. After threshold determination, target positive droplet concentration, as determined by QuantaSoft was normalized to RPP30 positive droplet concentration for that sample. RPP30 copy number was assumed to be equal to 2, as with previous studies. Copy number for target gene was thus determined as follows: $Target\;Copy\;Number=\frac{Target\;Positive\;Droplet\;Concentration}{RPP30\;Positive\;Droplet\;Concentration}*2.$ Technical duplicates were performed for every sample, and those with copy number reads greater than 20% apart were excluded from analysis. Unpaired non-parametric t-tests (Mann-Whitney tests) were performed for each comparison of copy numbers between conditions. Kruskall-Wallis tests were performed for each set of single organoids.

## Data Availability

The data presented in the study are deposited in the Zenodo repository, accession number 11099651.
