# Supplementary material for: Digital droplet PCR analysis of organoids generated from mouse mammary tumors demonstrates proof-of-concept capture of tumor heterogeneity
Source: Front Cell Dev Biol. 2024 May 15;12:1358583. doi: 10.3389/fcell.2024.1358583 (PMC11140600; doi:10.3389/fcell.2024.1358583)
Supplement: Supplementary file 1 [file DataSheet1.PDF]

Supplementary Material

Digital Droplet PCR analysis of organoids generated from mouse mammary tumors demonstrates proof-of-concept capture of tumor heterogeneity

Katherine E. Lake[1,2], Megan M. Colonnetta[2,3,4,5], Clayton A. Smith[1,2], Kaitlyn Saunders[1,2], Kenneth Martinez-Algarin[1,2], Sakshi Mohta[1,2], Jacob Pena[1,2], Heather L. McArthur[1,2], Sangeetha M. Reddy[1,2], Evanthia T. Roussos-Torres[6], Elizabeth H. Chen[2,3,4,5], and Isaac S. Chan[1,2,3,4]

[1]Department of Internal Medicine, Division of Hematology and Oncology, University of Texas Southwestern, Dallas, Texas, USA.

[2]Harold C. Simmons Comprehensive Cancer Center, University of Texas Southwestern Medical Center, Dallas, TX, USA.

[3]Department of Molecular Biology, University of Texas Southwestern, Dallas, Texas, USA.

[4]Hamon Center for Regenerative Science and Medicine, University of Texas Southwestern Medical Center, Dallas, TX, USA.

[5]Department of Cell Biology, University of Texas Southwestern Medical Center, Dallas, TX, USA.

[6]Division of Medical Oncology, Norris Comprehensive Cancer Center, Keck School of Medicine, University of Southern California, Los Angeles, CA, USA.

*** Correspondence:**
Isaac Chan, MD, PhD
Isaac.Chan@UTSouthwestern.edu

1 Supplementary Figures

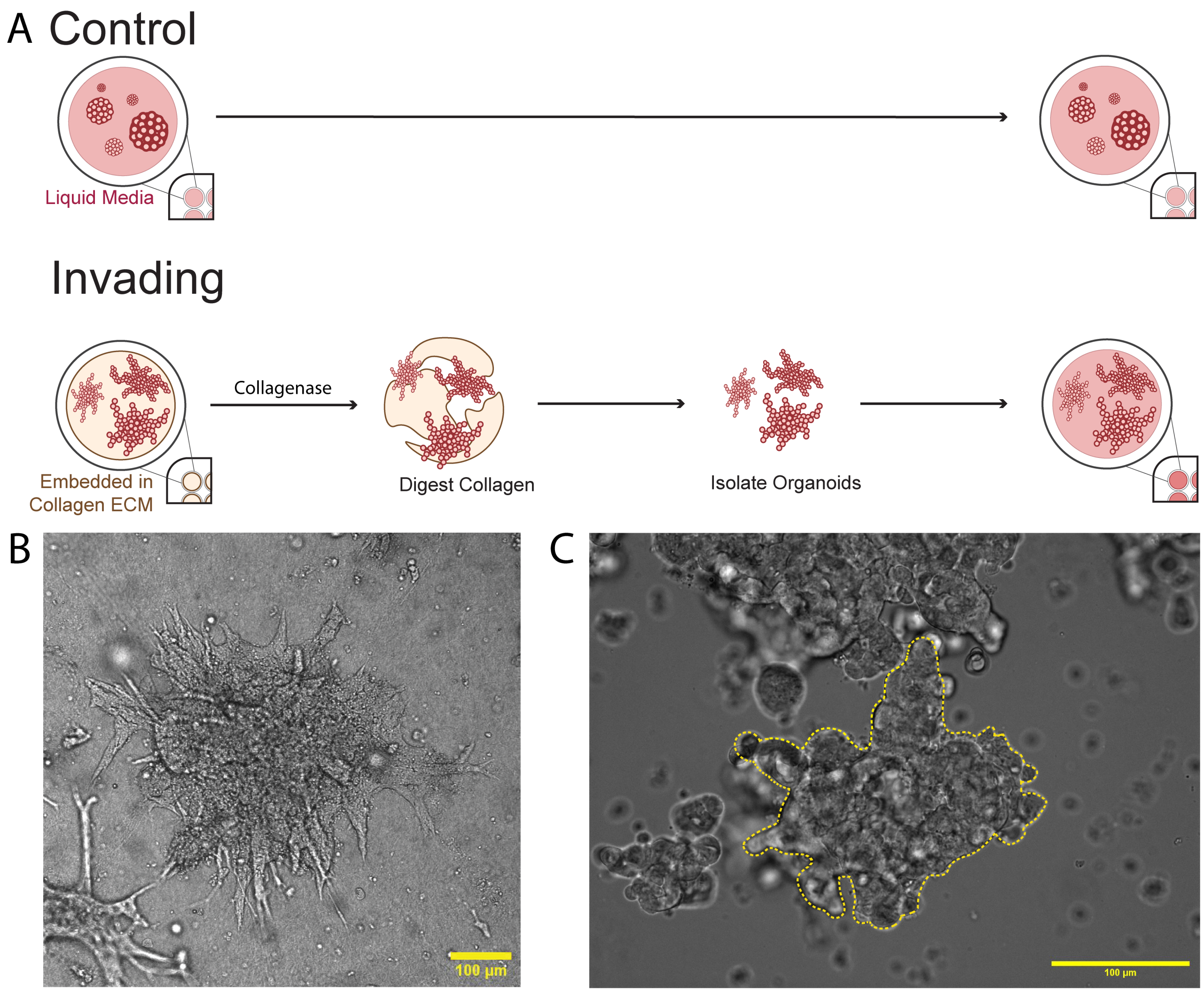

Supplemental Figure 1: Invading tumor organoids retain their morphology after isolation from ECM

(A) Schematic of organoid invasion assay for non-invading organoids (top) and invading organoids (bottom). Collagen was digested using collagenase for invading organoid conditions. Non-invading and invading organoids were pooled based on condition and gDNA isolated. **(B)** Mouse PyMT organoid embedded in collagen ECM. These organoids form invasive morphology in collagen ECM. **(C)** Representative image of an isolated invading organoids in liquid media, post-collagenase digestion. Invasive organoids retain their invasive morphology (outlined with yellow dotted line).

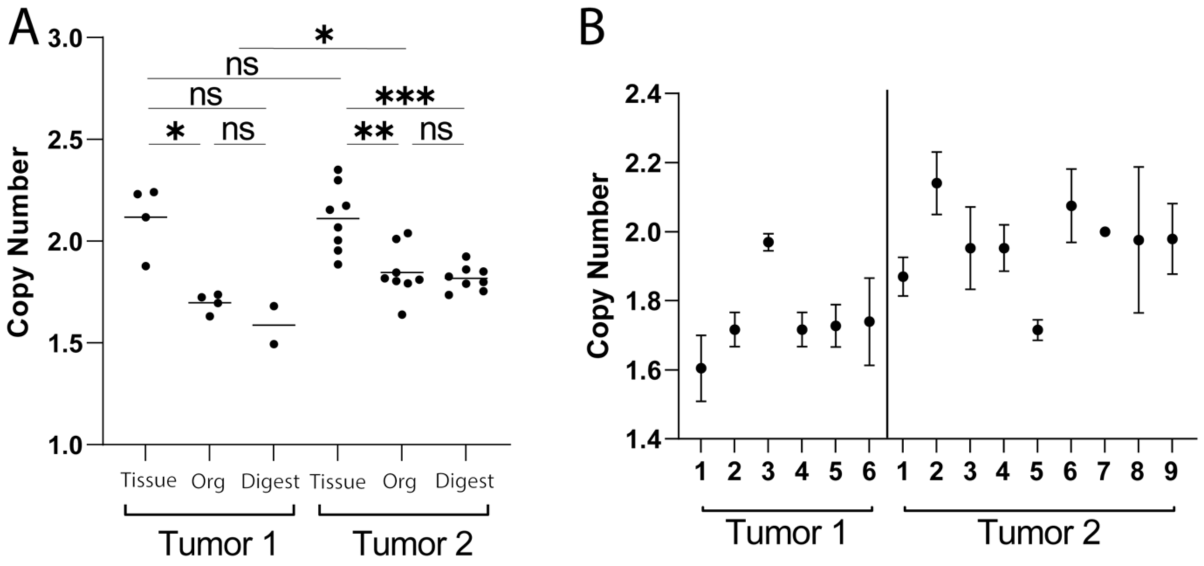

Supplemental Figure 2: Copy number of ADGRA2 in tissue, pooled organoids, single cell digests, and single organoids from mouse mammary tumors

(A) ADGRA2 copy numbers in tissue, pooled organoids (Org), and single cell digests (Digest) from two different mice. Tissue sample copy number alterations are statistically different than pooled organoid samples (Mann-Whitney, $p<0.05$). **(B)** ADGRA2 copy numbers in single organoids from tumors from two different mice.

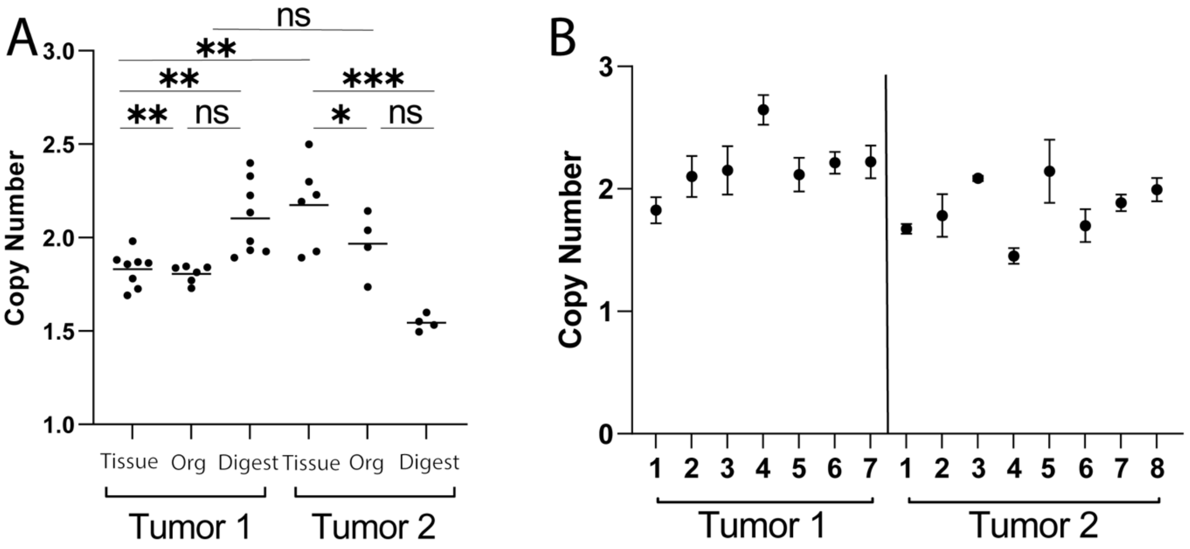

Supplemental Figure 3: Copy number of NSD3 in tissue, pooled organoids, single cell digests, and single organoids from mouse mammary tumors

(A) NSD3 copy numbers in tissue, pooled organoids (Org), and single cell digests (Digest) from two different mice. Tissue sample copy number alterations are statistically different than pooled organoid samples (Mann-Whitney, $p<0.05$). **(B)** NSD3 copy numbers in single organoids from tumors from two different mice.

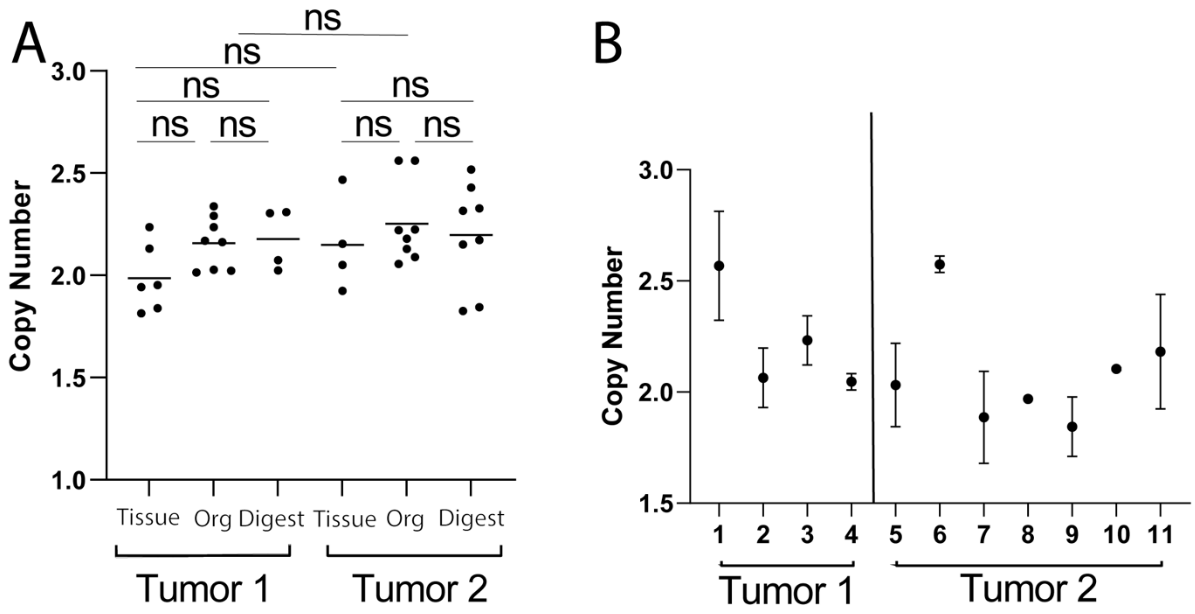

Supplemental Figure 4: Copy number of PAK1 in tissue, pooled organoids, single cell digests, and single organoids from mouse mammary tumors

(A) PAK1 copy numbers in tissue, pooled organoids (Org), and single cell digests (Digest) from two different mice. No statistically significant differences in PAK1 copy number were appreciated (Mann-Whitney). **(B)** PAK1 copy numbers in single organoids from tumors from two different mice.
